# The IgG-specific endoglycosidases EndoS and EndoS2 are distinguished by conformation and antibody recognition

**DOI:** 10.1016/j.jbc.2024.107245

**Published:** 2024-04-01

**Authors:** Abigail S.L. Sudol, Max Crispin, Ivo Tews

**Affiliations:** School of Biological Sciences, University of Southampton, Southampton, UK

**Keywords:** antibody, glycosylation, crystal structure, endoglycosidase, enzyme, Fc, glycoside hydrolase, immunoglobulin G

## Abstract

The IgG-specific endoglycosidases EndoS and EndoS2 from *Streptococcus pyogenes* can remove conserved *N*-linked glycans present on the Fc region of host antibodies to inhibit Fc-mediated effector functions. These enzymes are therefore being investigated as therapeutics for suppressing unwanted immune activation, and have additional application as tools for antibody glycan remodeling. EndoS and EndoS2 differ in Fc glycan substrate specificity due to structural differences within their catalytic glycosyl hydrolase domains. However, a chimeric EndoS enzyme with a substituted glycosyl hydrolase from EndoS2 loses catalytic activity, despite high structural homology between the two enzymes, indicating either mechanistic divergence of EndoS and EndoS2, or improperly-formed domain interfaces in the chimeric enzyme. Here, we present the crystal structure of the EndoS2-IgG1 Fc complex determined to 3.0 Å resolution. Comparison of complexed and unliganded EndoS2 reveals relative reorientation of the glycosyl hydrolase, leucine-rich repeat and hybrid immunoglobulin domains. The conformation of the complexed EndoS2 enzyme is also different when compared to the earlier EndoS-IgG1 Fc complex, and results in distinct contact surfaces between the two enzymes and their Fc substrate. These findings indicate mechanistic divergence of EndoS2 and EndoS. It will be important to consider these differences in the design of IgG-specific enzymes, developed to enable customizable antibody glycosylation.

The pathogen *Streptococcus pyogenes* belongs to the gram-positive group A streptococci (GAS) that can cause mild diseases such as skin infection but also life-threatening systematic illness (1). The bacterium is highly adapted to human infection and has developed a range of immune evasion mechanisms for prolonged infection (2). In particular, *S. pyogenes* possesses protease and glycosyl hydrolase enzymes to specifically target and degrade immunoglobulin G (IgG) antibodies, the most abundant antibody class within human serum (3). The IgG-degrading enzyme of *S. pyogenes* (IdeS) is a protease that deactivates IgG by cleaving within the lower hinge region, yielding F(ab′)_2_ and Fc fragments (4, 5), while the protease SpeB cleaves a broader range of immunoglobulins as well as other components of the immune system (6, 7). Streptococcal Endoglycosidase S (EndoS) (8) and Endoglycosidase S2 (EndoS2) (9) enzymes remove *N*-linked glycans from IgG at N297 within the Fc Cγ2 domain that are conserved across all IgG subtypes (10).

Glycosylation of the Fc domain is implicated in maintaining Fc structural integrity (11, 12, 13, 14, 15, 16) and Fc gamma receptor (FcγR) and complement-mediated immune activation (3, 14, 17); thus, removal of the glycan impedes Fc-mediated effector functions (16, 18). Increased virulence and survival of the bacterium *in vivo* was found to be due to IgG glycan hydrolysis by EndoS (19). Moreover, improved survival of *S. pyogenes* in an opsonophagocytic assay was shown to relate to reduced FcγR- and complement-mediated immune activation (20). Promising results have been reported from preclinical models of autoimmune disease that successfully utilized EndoS treatment for inactivation of pathogenic antibodies (21, 22, 23, 24, 25, 26, 27, 28, 29, 30); combination with IdeS additionally showed inactivation of donor-specific antibodies in a murine model of bone marrow transplantation (31). The specific deactivation of competing serum IgG by EndoS and/or IdeS is also being investigated for the potentiation of therapeutic antibodies (16, 32).

EndoS and EndoS2 are additionally used as tools for antibody glycoengineering (33, 34). They can remove unwanted glycosylation or generate more desired glycoforms when combined with transglycosidases (35, 36, 37, 38, 39). Such transglycosylation reactions have been optimized using enzyme variants, although both wild-type EndoS (40, 41) and EndoS2 (37) possess some transglycosylation activity. A further development has been the use of wild-type EndoS2 in “one-step” reactions for the synthesis of antibody-drug conjugates (42, 43). The precise control of antibody glycosylation has been applied in several clinically used antibodies for improved immune effector function (44, 45, 46, 47, 48), demonstrating their utility in antibody glycoengineering.

The glycosyl hydrolases EndoS and EndoS2 cleave the β1−4 glycosidic linkage between the first two *N*-acetylglucosamine (GlcNAc) saccharide residues to shed the main portion of the glycan, leaving a single GlcNAc variably modified with α1−6 fucose. Although they possess a similar domain architecture, EndoS and EndoS2 differ in specificity towards IgG Fc glycans. While EndoS is specific towards complex-type, biantennary glycans (8, 40), the EndoS2 enzyme exhibits broader specificity, additionally cleaving less abundant classes of Fc *N*-linked glycans, such as the oligomannose-type that bears an extra antenna compared to biantennary substrates, and the hybrid-type glycans that are composed of two mannose-terminating antennae alongside a complex-type branch (9, 49). The underlying mechanism of glycan substrate binding is well understood from structural studies, demonstrating how active site loops 1, 6, and 7 define the binding of the pentasaccharide core in both enzymes. The key differences occur in the structures of loops 4 and 5 (50, 51): the extra space in the glycan binding pocket within EndoS2 provides a structural rationale for the biochemically characterized broader glycan substrate specificity exhibited over EndoS.

EndoS and EndoS2 are multi-domain enzymes, comprised of a catalytic glycosyl hydrolase (GH) domain, a leucine-rich repeat (LRR) domain, a hybrid immunoglobulin fold (hIg) domain, and the so-called carbohydrate-binding module (CBM) (50, 52, 53, 54); EndoS additionally has N- and C-terminal 3-helix bundles (50, 53). Recent structural studies revealed that the functional role of the CBM in EndoS was not to bind carbohydrates but rather to specify peptide binding to the Fc surface (55, 56). The structural basis for IgG recognition by EndoS2 is less clear, although hydrogen-deuterium exchange data show a role for both GH and CBM domains in Fc binding (51), which indicates a similar mode of Fc recognition to that observed for EndoS (55, 56).

EndoS and EndoS2 are highly specific for IgG antibodies (8, 9), and therefore have the potential to be exploited for the design of IgG-specific chimeric enzymes, with substituted catalytic domains to enable customizable IgG Fc glycosylation. However, experiments to manipulate EndoS substrate specificity by creation of a chimeric enzyme containing a substituted catalytic domain from EndoS2 have led to a non-functional enzyme (51), which raises a number of hypotheses. It is possible that interdomain interfaces may not form correctly in the chimeric enzyme; additonally, the Fc-contacting GH and CBM domains may not be positioned correctly in the chimeric enzyme. Klontz *et al.* found that substitution of both EndoS2 GH and CBM produced an active EndoS2-like enzyme (albeit with lower activity than wild-type EndoS2), indicating that each CBM must work in tandem with its matching catalytic domain (51), which lends some support to the second hypothesis.

Here, we present the crystal structure of EndoS2 in complex with IgG1 Fc, with the motivation of rationalizing these previous biochemical data. While the basis for Fc recognition by EndoS2 was previously unclear, the structural data presented here now reveals the different orientations of EndoS2 towards the substrate compared with EndoS, providing evidence for distinct modes of substrate recognition by these two enzymes.

## Results

### Structure of the EndoS2/IgG1-Fc complex

**Figure 1:**
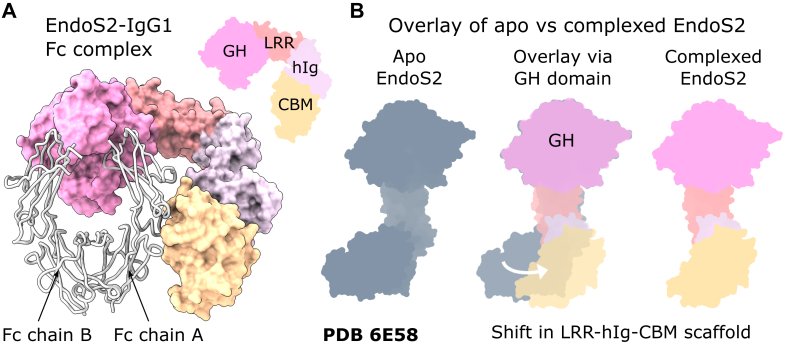
**Structure of EndoS2**^**D184A/E186L**^**-IgG1 Fc complex.***A*, overall structure of the EndoS2-Fc complex, with the second copy of EndoS2 omitted for clarity (compare to Fig. S1). IgG1 Fc is coloured silver, and EndoS2 domains are coloured as follows: glycosyl hydrolase (GH, *pink*, residues 43–386); leucine-rich repeat (LRR, *coral*, residues 387–547); hybrid immunoglobulin (hIg, *lilac*, residues 548–680); carbohydrate-binding module (CBM, *yellow*, residues 681–843). *B*, the superposition of EndoS2 from the IgG1 Fc complex with unliganded EndoS2 (PDB 6E58 (51)) based on the GH domain reveals a displacement of the LRR–hIg–CBM scaffold.

The crystal structure of inactive EndoS2 (containing D184A/E186L substitutions to abolish catalytic activity) in complex with IgG1 Fc substrate was determined by X-ray crystallography to a resolution of 3.0 Å (Table S1). E382A substitutions were introduced into the Fc to discourage crystallization of the Fc only, as previously utilized  (55). As also observed in the earlier EndoS-Fc complex (55), two EndoS2 enzymes are bound to a single Fc homodimer (Fig. S1). Figure 1 illustrates how the EndoS2 enzyme interacts with the Fc fragment. The enzyme captures a single Cγ2 domain of the Fc through contact with the GH domain and the CBM domain.

Comparison of the structure of EndoS2 complexed with Fc as determined here with an earlier structure of the unliganded EndoS2 enzyme (PDB 6E58 (51)) reveals a different positioning of the four EndoS2 domains (Fig. 1*B*). When aligning the enzyme structures based on the GH domain (using Cα positions for amino acids 46–386), the adjacent LRR is offset by 10°, which leads to displacement of the hIg and CBM domains (with RMSDs of 8.2 Å and 19.4 Å, respectively, Fig. 1*B*). Superposition based on the CBM (using Cα positions for amino acids 681–843) results in slightly smaller domain shifts: the adjacent hIg domain is tilted by 4.2°, while the LRR and GH are displaced by 5.1 Å and 6.3 Å, respectively (Fig. S2*A*). The different positioning of the GH and CBM domains is supported by rearrangement of the LRR–hIg scaffold, as evident from superposition based on the LRR domain (using Cα positions for amino acids 387–547, Fig. S2*B*), which reveals an 11.6° tilt in the hIg. In comparison, no changes in domain orientation were observed in the unliganded EndoS2 enzyme upon glycan binding (51).

The structural comparison of bound and free enzymes demonstrates that the arrangement of the four domains is not rigid, and in particular, shows that the GH, LRR, and hIg domains have some freedom to reorient relative to each other, allowing EndoS2 to sample a number of conformations. We have investigated this by comparing the three copies of the EndoS2-Fc gamma chain complex contained in the asymmetric unit, which each display different *B* factors (see Fig. S3 and Table S2).

While the analysis supports slightly different conformations for all three copies, they are all consistently different from the free enzyme (Fig. S4). In addition, the conformation of the individual domains is identical in all copies and when compared to unliganded EndoS2 (Table S3). Thus, domain rearrangements in EndoS2 appear to be required for Fc binding.

**Figure 2:**
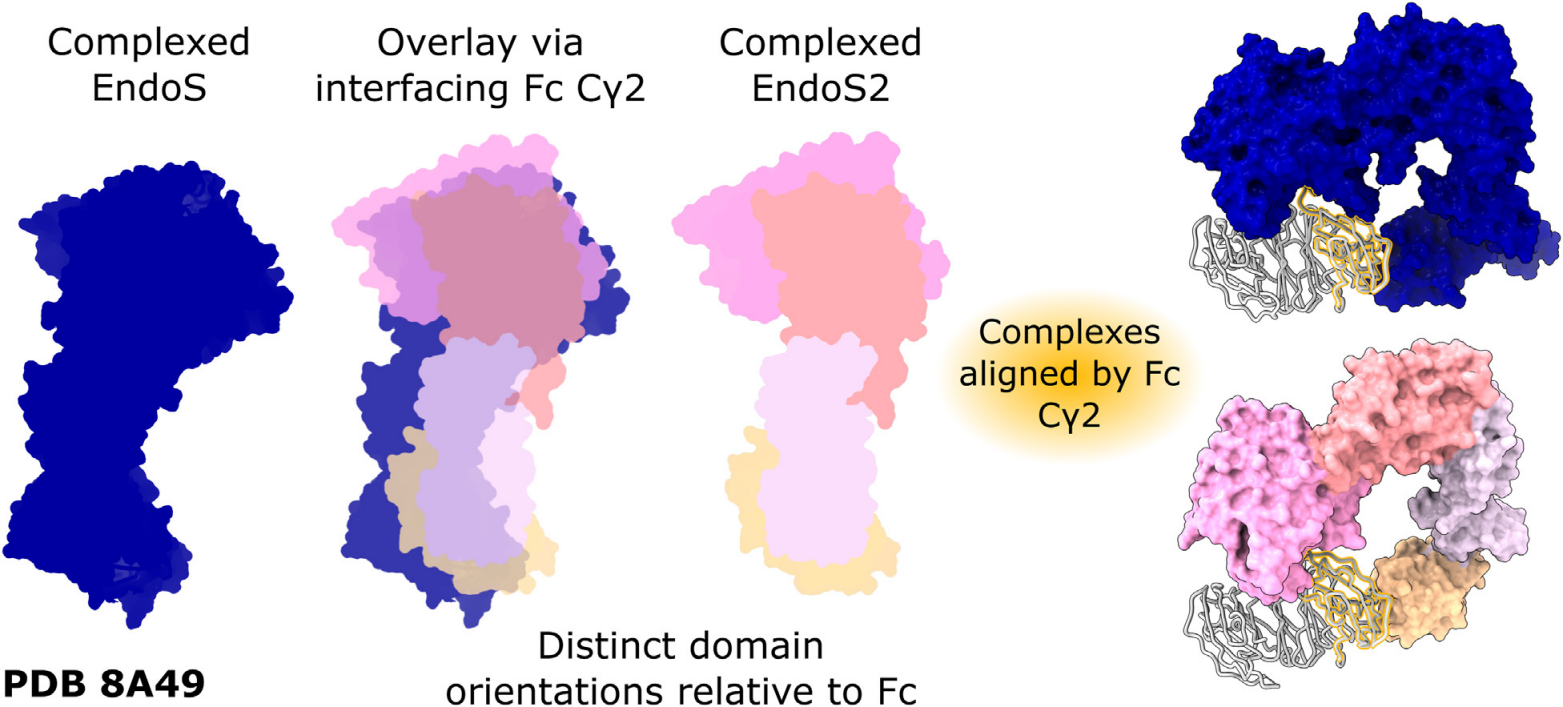
**Distinct modes of capture of IgG1 Fc by EndoS2 and EndoS.** Superposition of the two complexes based on the Fc Cγ2 domain (using Cα positions of amino acids 237–340) reveals a different orientation of the EndoS2/EndoS GH and CBM domains relative to the Fc. EndoS2 domains are colored as in Figure 1; EndoS is colored *dark blue*; IgG1 Fc is colored *silver*, with the interfacing Fc Cγ2 domain highlighted in *gold*. The N-terminal proline-rich loop and C-terminal 3-helix bundle (comprising amino acids 98–112 and 924–998, respectively) within EndoS (PDB 8A49 (55)) are omitted for clarity.

We next compared the EndoS2 complex with the earlier determined structure of the EndoS-IgG1 Fc complex (PDB 8A49 (55)) to understand the difference in Fc substrate specificity (Fig. 2). Superposition used the interfacing Fc Cγ2 domain as reference (using Cα positions of amino acids 237–340) and revealed different orientation of the GH and CBM domains within EndoS and EndoS2, which is also accompanied by different positioning of LRR and hIg domains (Figs. 1*B* and 2).

The different capture of the Fc Cγ2 domain by the two enzymes is accompanied by a more “open” conformation of the Fc fragment, with Cγ2 domains further apart in the EndoS2-Fc complex (Fig. S5*A*). This is possibly due to the GH domain contact surface protruding more into the space between the Fc Cγ2 domains in the EndoS2 complex, relative to that in EndoS (Fig. S5*B*). Thus, EndoS2- and EndoS-Fc complexes capture the Fc Cγ2 domains in different orientation.

### Fc peptide recognition by the EndoS2 CBM

To understand the functional consequence of the different orientation of the EndoS2 enzyme, we investigated the contact interfaces between EndoS2 and the IgG1 Fc Cγ2 domain and compared this with the interaction identified in the EndoS complex. Both EndoS2 and EndoS similarly contact one Fc Cγ2 domain *via* their GH and CBM domains. Contact areas are similar, but smaller for the EndoS2-Fc complex. The overall EndoS2-IgG1 Fc interface has a surface area of 978 Å^2^ and a solvation-free energy gain of −8.6 kcal/mol, while EndoS forms a 1324 Å^2^ interface, with a −9.1 kcal/mol solvation-free energy gain as calculated with PDBePISA (57).

**Figure 3:**
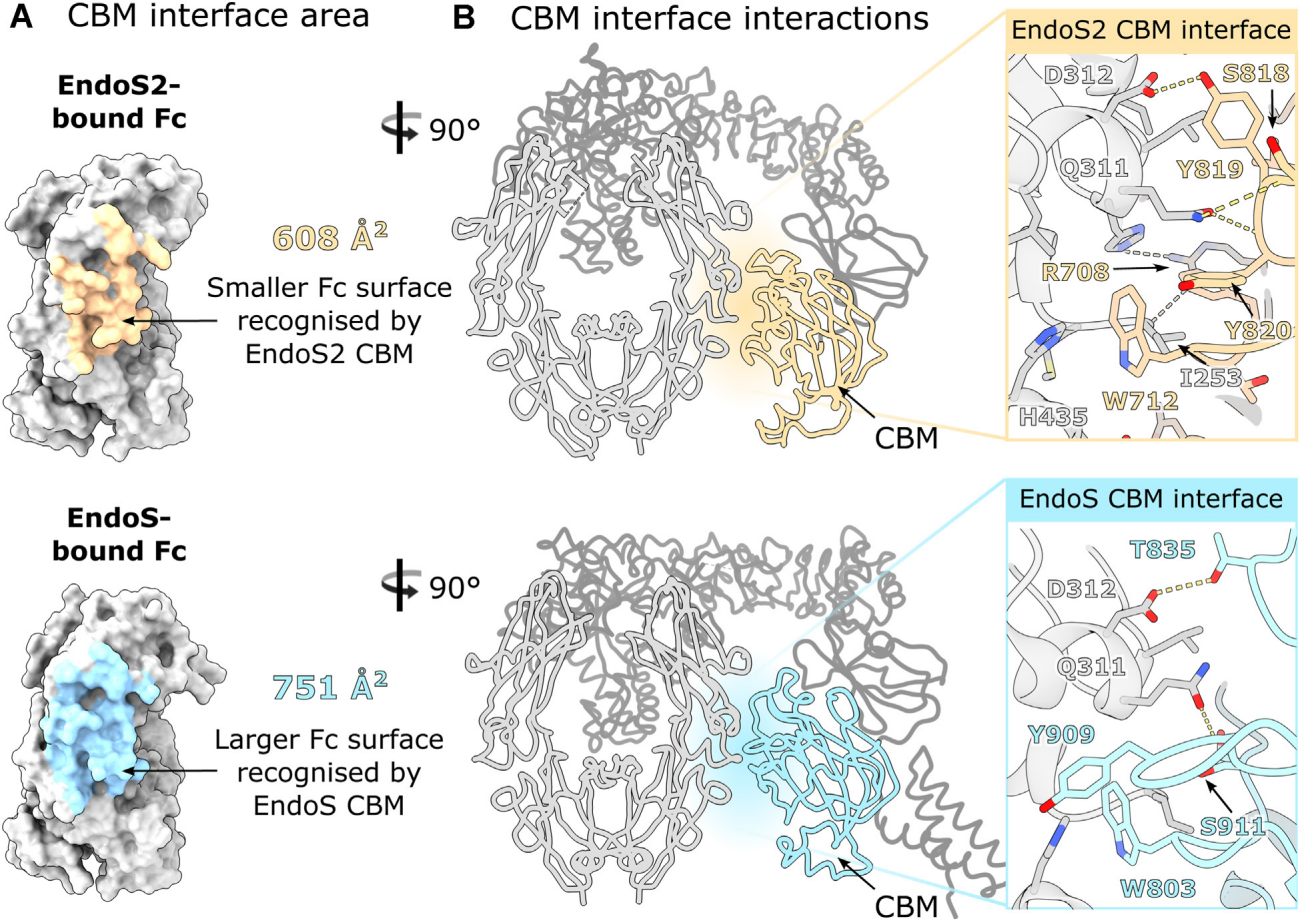
**Comparison of EndoS2/EndoS CBM interface with IgG1 Fc.***A*, interface area of the EndoS2/EndoS CBM and Fc, as calculated by PDBePISA (57), shown in *yellow* for EndoS2 and in blue for EndoS, respectively. *B*, interactions present at the EndoS2/EndoS CBM interface. IgG1 Fc is colored *silver*; EndoS2/EndoS are colored *dark grey* with their CBMs colored in *yellow* and *blue*, respectively. Hydrogen bonds are depicted as *yellow dashes*. Interacting Fc/EndoS2 amino acids are shown as *sticks* and colored by *heteroatom* (oxygen, *red*; nitrogen, and *blue*).

The interface of the CBM with the Fc fragment was compared between the EndoS2 and EndoS complexes (Fig. 3). While both CBMs contact the Fc at the Cγ2–Cγ3 boundary, the interface of 608 Å^2^ in EndoS2 is smaller compared to the 751 Å^2^-sized interface formed by EndoS (Fig. 3*A*). While interactions between enzyme and Fc are similar, the interfacing residues are not strictly conserved (Fig. 3*B*).

Amino acid W712 in EndoS2 has been shown to be important for Fc binding, as a substitution to alanine abolished hydrolytic activity towards IgG Fc bearing complex-type or oligomannose-type glycans (51). The EndoS2-Fc structure reveals how W712 binds within a hydrophobic pocket at the Fc Cγ2–Cγ3 interface, formed by I253, H310, L314, N434, and H435. Thus, W712 makes comparable interactions to W803 in EndoS, which was similarly shown to be essential for enzyme activity (53) and was observed binding in the same cavity within the EndoS-Fc crystal structure (55) (Fig. 3*B*). Another important amino acid in EndoS2 is Y820: Klontz *et al.* found that a serine exchange variant of this side chain severely reduced hydrolytic activity towards IgG bearing both complex- and oligomannose-type glycans (51). This side chain makes not only a main chain contact with Fc residue Q311 but also forms stacking interactions with the Q311 side chain. The equivalent amino acid in EndoS is a serine, similarly providing a main chain interaction with Q311 but lacking the hydrophobic interaction (55). Lastly, the side chain of EndoS2 Y819 hydrogen bonds to the D312 side chain from the Fc, an interaction fulfilled by the T835 side chain in EndoS (55). Comparison of the EndoS2 and EndoS CBM interfaces with IgG1 Fc reveals some unique interactions formed by EndoS2, such as the side chain of R708 that hydrogen bonds to the Fc backbone (Fig. 3*B*). These subtle differences play to the similar but not identical recognition of the Fc peptide by the two enzymes.

### Carbohydrate recognition by the GH domain

**Figure 4:**
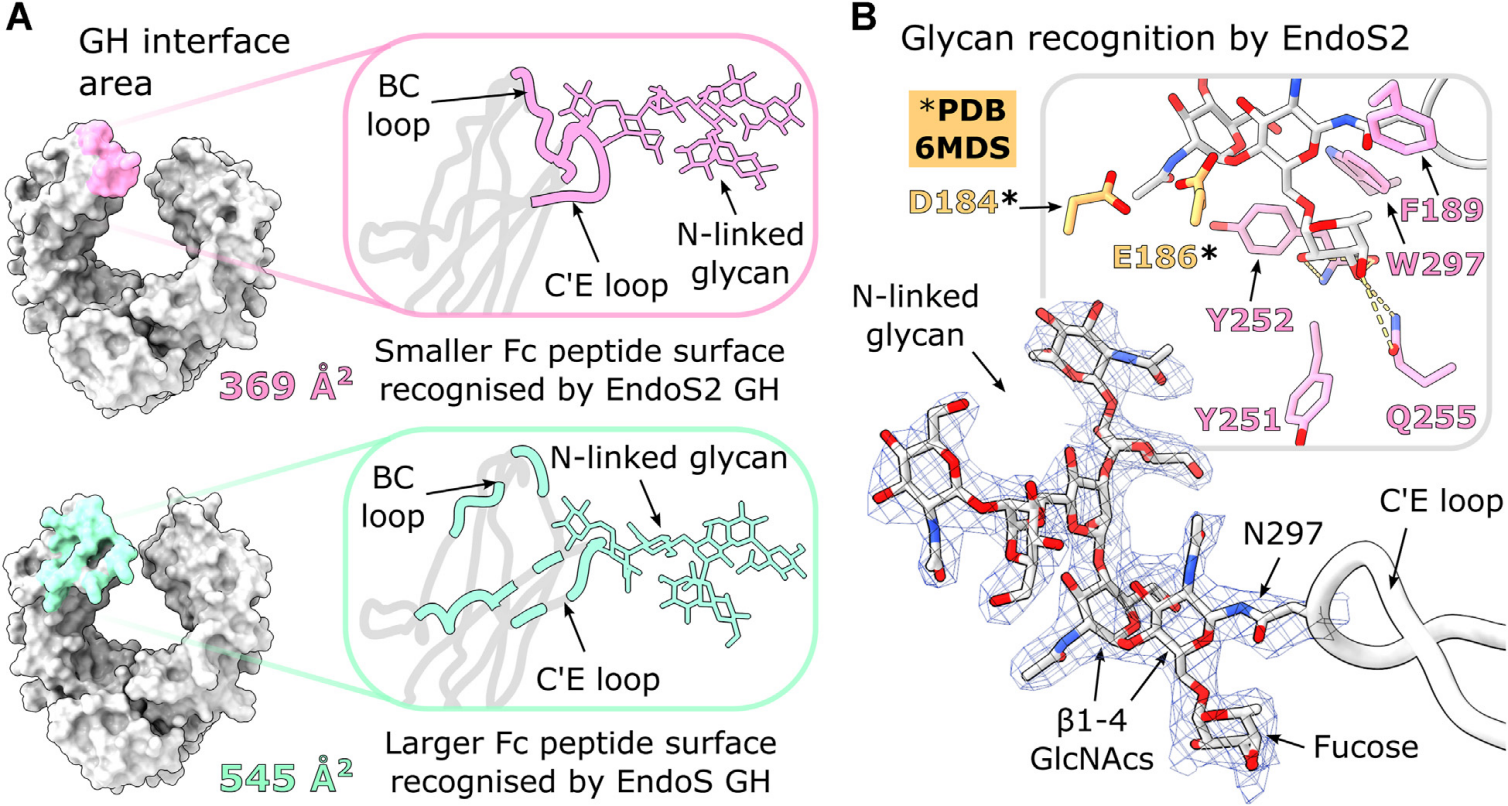
**Comparison of Fc peptide and glycan recognition by the EndoS2/EndoS GH domain.***A*, interface area of the EndoS2/EndoS GH interacting with the Fc, as calculated by PDBePISA (57), with the Fc contact surface colored in *pink* and *cyan*, respectively. *B*, binding of the uncleaved Fc *N*-linked glycan in the EndoS2 active site. The approximate positions of catalytic dyad residues D184 and E186 are indicated (exchanged to alanine and leucine in our structure, respectively). The superposition with wild-type EndoS2 (PDB 6MDS (51)) is based on the GH domain. The final 2*F*_obs_ − *F*_calc_ electron density map is shown at 1.5*σ* for the Fc *N*-linked glycan and for residue N297. IgG1 Fc is coloured *silver*; EndoS2 residues are coloured *pink*. Hydrogen bonds are depicted as *yellow dashes*. Fc residue N297, *N*-linked glycans and EndoS2 residues are shown as sticks and coloured by *heteroatom* (oxygen, *red*; nitrogen, *blue*).

Similar to the interaction of the CBM, the GH domain makes a smaller interface with the Fc in the EndoS2 complex (369 Å^2^) compared to the EndoS complex (545 Å^2^). This largely arises from a greater contact surface with the Fc C′E loop bearing the *N*-linked glycan in the EndoS complex (Fig. 4*A*). However, both enzymes display a similar mode of Fc glycan capture: superposition of the interfacing Fc Cγ2 (using Cα positions of amino acids 238–340) with respect to a wild-type Fc (PDB 3AVE) reveals that conformational differences are mainly limited to the C′E loop (Fig. S6). As also seen within the EndoS complex (55), the Fc glycan is observed in a “flipped-out” state (Fig. S6) and is well-ordered within the crystal structure (Fig. S7). In contrast, the glycans within Fc crystal structures are typically observed sitting in between the Cγ2 domains and interacting with Fc surface residues (10, 58, 59, 60).

Crystal structures of EndoS2 in complex with oligomannose- and complex-type glycan substrates have previously identified GH amino acids involved in glycan binding (51). The EndoS2-Fc complex structure additionally reveals interactions with the first GlcNAc and fucose moieties within the glycan. The aromatic side chains of F189, Y251, Y252, and W297 provide hydrophobic stacking interactions, while the Y252 backbone and Q255 side chains form hydrogen bonds with the fucose (Fig. 4*B*).

We observe an atypical Φ dihedral torsion angle of 5.9° for the β1–4 glycosidic linkage between the two core GlcNAcs. This value lies outside the range of average GlcNAc β1–4 GlcNAc linkages reported from crystal structures (−74° ± 8.4, (61)). The distortion is consistent with the formation of a higher energy state that promotes cleavage of the β1–4 bond. Such glycan distortion is also observed within the EndoS-Fc complex, with an equivalent Φ torsion angle of −59° between the two core GlcNAcs (55). In addition, several crystal structures of endoglycosidases in complex with their cleaved glycan substrate show the second GlcNAc adopting a higher-energy, skew-boat conformation presumably having the effect of lowering the energy of the transition state (51, 62, 63). The second GlcNAc is bound close to the catalytic dyad residues D184 and E186 (exchanged to alanine and leucine in our structure, respectively; Fig. 4*B*). An overlay with the wild-type enzyme (PDB 6MDS) reveals that E186 would be oriented towards the distorted β1–4 linkage, and thereby positioned for nucleophilic attack and subsequent cleavage of the glycan.

## Discussion

The EndoS2-IgG1 Fc crystal structure demonstrates how the enzyme contacts IgG1 Fc with its GH and CBM domains, as also elucidated from hydrogen-deuterium exchange of an EndoS2-rituximab complex (51) and similarly observed for the related EndoS complex (55, 56). However, upon binding to the Fc, EndoS2 undergoes a domain rearrangement, with the relative orientation of GH, LRR, and hIg domains changing (Figs. 1 and S2). A molecular dynamics study by Aytenfisu *et al.* (54) predicted a range of conformational states adopted by EndoS2, measured by the smallest inter-domain distance between the GH and CBM domains. Such distances were reported to vary between “open” states, as observed in the unliganded enzyme (PDB 6E58, ∼50 Å), and “closed” (less than 20 Å). In complexed EndoS2, this distance is ∼25 Å, giving experimental validation of the earlier simulation and revealing the exact conformation of the closed enzyme state. EndoS2 domain flexibility is also observed when comparing the three copies of the EndoS-Fc complex within the asymmetric unit (Fig. S4). Our crystal structure therefore indicates that flexibility and reorientation of CBM and GH against the central scaffold is an essential property of the EndoS2 enzyme; this is in contrast to the EndoS enzyme, which was not observed undergoing large domain rearrangements upon Fc binding (55).

We show how the EndoS2 enzyme orients its Fc-contacting GH and CBM domains differently with respect to the Fc when compared to the earlier EndoS-Fc complex (Fig. 2). This coincides with distinct regions of the Fc peptide surface being targeted by the GH and CBM within the two enzymes (Figs. 3 and 4). A sequence alignment of EndoS and EndoS2 additionally reveals that side chains interfacing with the Fc protein are not particularly conserved (Fig. S8). Moreover, aside from residue W712 (W803 in EndoS), other CBM residues found to be important for IgG Fc binding are not conserved (R908 and E833 in EndoS (53); Y820 in EndoS2 (51)). These observations indicate distinct modes of IgG recognition by the two endoglycosidases.

The observed differences in the binding of EndoS and EndoS2 to their IgG1 Fc substrate help to rationalize previous experiments with chimeric EndoS/EndoS2 enzymes. It was shown that, despite high structural homology between EndoS and EndoS2, a simple substitution of the EndoS2 GH onto an EndoS scaffold was not sufficient to confer EndoS2-like activity, and instead produced an inactive enzyme. However, the substitution of both EndoS2 GH and CBM domains could convert enzymatic activities, posing the question of how this can be understood and subsequently utilized in the design of IgG-specific endoglycosidases (51). One explanation for this would be improperly-formed interdomain interfaces within the chimeric enzyme. A sequence alignment of EndoS and EndoS2 (Fig. S9) shows how GH residues that interface with the respective LRR domains are partially, but not fully, conserved, which may result in sub-optimal positioning of Fc-contacting domains within the chimeric enzymes. However, the altered interdomain interfaces also support the distinct EndoS/EndoS2 domain orientations relative to the Fc observed within the crystal structure. Moreover, catalytic activity demonstrated by the enzyme with both GH and CBM substituted (51) indicates that the altered domain interfaces are not significantly impeding activity.

An alternative hypothesis is that the orientation of Fc-contacting CBM and GH domains relative to the enzyme scaffold may not lead to an active conformation in the chimeric enzyme. As discussed above, our EndoS2-Fc crystal structure shows how EndoS and EndoS2 interface with distinct regions of the Fc peptide surface. This suggests that a substituted EndoS2 GH is not positioned correctly to bind its Fc substrate (with respect to the remaining EndoS scaffold), and thus lends credence to this second hypothesis. Alterations in the LRR–hIg scaffold as observed within the crystal structure (Figs. 1*B* and S2*B*) are likely also required for optimal enzymatic activity, which rationalizes the previous observation that a chimeric enzyme with both GH and CBM substituted was not fully active when compared to wild-type EndoS2 (51).

A recent paper by Fan *et al.* demonstrated how the glycosyl hydrolase domains in EndoS and EndoS2 can be replaced with an α-L-fucosidase from *Lactobacillus casei* BL23 for significantly enhanced antibody defucosylation, when compared to the native fucosidase (64). In this work, various constructs of both EndoS and EndoS2 with the substituted fucosidase domain were tested (64). We envisage that the EndoS2-Fc crystal structure presented here can aid in future efforts to design such IgG-specific glycosidases and glycosyltransferases, by providing a structural model of the EndoS2 enzyme complexed with Fc substrate, onto which potential glycosidase/glycosyltransferase domains can be modeled.

To conclude, crystallographic analysis of EndoS2 in complex with its IgG1 Fc target not only reveals a fundamental immune evasion mechanism of the *S. pyogenes* bacterium but can also aid future efforts in designing IgG-specific endoglycosidases for use in engineering antibody glycosylation.

## Experimental procedures

Detailed materials and methods used for cloning, protein expression and purification, crystallization, and structure determination of the EndoS2-IgG1 Fc complex are included within the supporting information.

## Data availability

Model coordinates and structure factors for the EndoS2^D184A/E186L^-IgG1 Fc^L234C/E382A^ crystal structure have been deposited in the Protein Data Bank with accession number 8Q5U.

## Supporting information

This article contains supporting information (51, 55, 57, 65, 66, 67, 68, 69, 70, 71, 72, 73, 74, 75, 76, 77).

## Conflict of interest

The authors declare that they have no conflicts of interest with the contents of this article.
