# Supplementary material for: The IgG-specific endoglycosidases EndoS and EndoS2 are distinguished by conformation and antibody recognition
Source: J Biol Chem. 2024 Apr 1;300(5):107245. doi: 10.1016/j.jbc.2024.107245 (PMC11063906; doi:10.1016/j.jbc.2024.107245)
Supplement: Supporting Information [file mmc1.pdf]

Supporting information for:

The IgG-specific endoglycosidases EndoS and EndoS2 are distinguished by conformation and antibody recognition

Abigail S. L. Sudol[1], Max Crispin[1]*, Ivo Tews[1]*.

[1]School of Biological Sciences, University of Southampton, Southampton, SO17 1BJ, UK

*Correspondence to Ivo Tews (Ivo.Tews@soton.ac.uk) and Max Crispin (Max.Crispin@soton.ac.uk)

Table S1: Crystallographic data collection and refinement statistics for EndoS2$^{D184A/E186L}$-IgG1 Fc$^{L234C/E382A}$ complex. Values for the highest resolution shell are reported in parentheses.

**Data Collection**	
Beamline	I03 (Diamond Light Source)
Resolution range (Å)	49.91–3.00 (3.05–3.00)
Space group	$P4_32_12$
Unit cell dimensions:<br>$a = b, c$ (Å)<br>α, β, γ (°)	 228.8, 161.6<br>90, 90, 90
Wavelength (Å)	0.9763
Unique reflections	85,926 (4,250)
Completeness (%)	100 (100)
R_{merge}	0.187 (2.614)
R_{meas}	0.191 (2.668)
R_{pim}	0.036 (0.527)
$I/\sigma(I)$	12.6 (0.5)
Multiplicity	27.5 (25.4)
$CC_{1/2}$	1.000 (0.640)
Wilson B factor (Å^2)	77
**Refinement**	
Number of reflections (all / free)	85,849 / 4,376
R_{work} (%)	21.6
R_{free} (%)	25.2
RMSD[1]:<br>Bonds (Å)<br>Angles (°)	 0.0066<br>1.18
Molecules per ASU[2]	6 (3 EndoS2 chains / 3 Fc gamma chain fragments)
Atoms per ASU[2]	21,168
Average B factors (Å^2) protein / ligand / carbohydrate / water	105.6 / 113.0 / 100.7 / 73.1
Model quality (Ramachandran plot)[3]:<br>Most favoured region (%)<br>Allowed region (%)<br>Outliers (%)	 96.06<br>3.75<br>0.19, 0.0[4]

[1] RMSD, root-mean-squared deviation
[2] ASU, asymmetric unit
[3] as calculated with Molprobity (1)
[4] as reported by the wwPDB Structure Validation Report

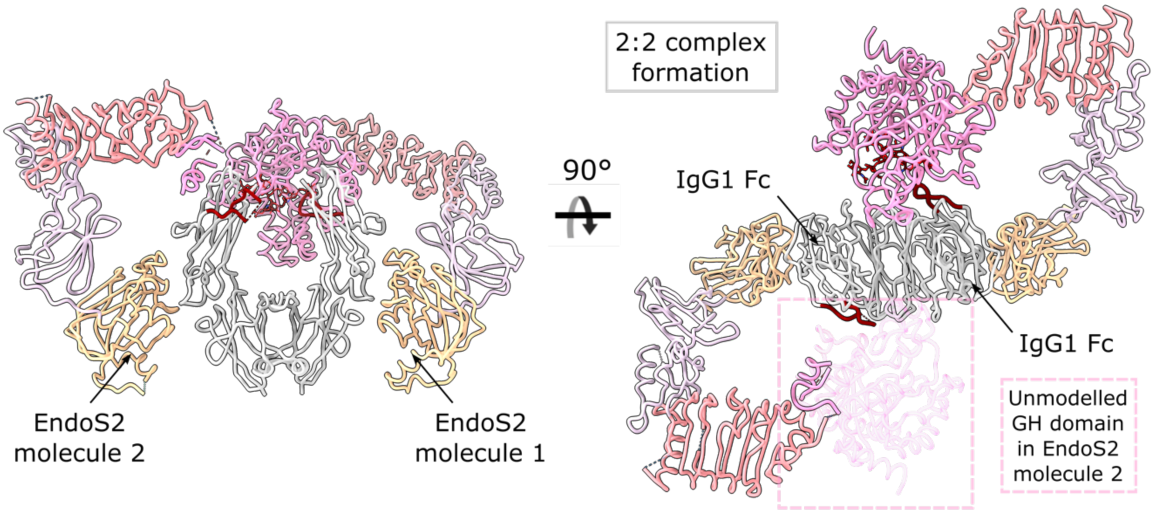

Figure S1: The 2:2 stoichiometry of the EndoS2$^{D184A/E186L}$-IgG1 Fc$^{L234C/E382A}$ complex. In the crystal structure, one EndoS2 molecule is seen in contact with each IgG1 Fc γ-chain. As each Fc is a homodimer of γ-chains, a 2:2 stoichiometry results, similar to what was seen in the EndoS-IgG1 Fc complex (2). The electron density for the GH domain is weak for one of the EndoS2 copies and consequently was not included in the model (faint pink model as indicated from superposition of the two EndoS2 molecules). EndoS2 and IgG1 Fc are coloured as in Figure 1; Fc C′E loops and *N*-linked glycan are coloured maroon.

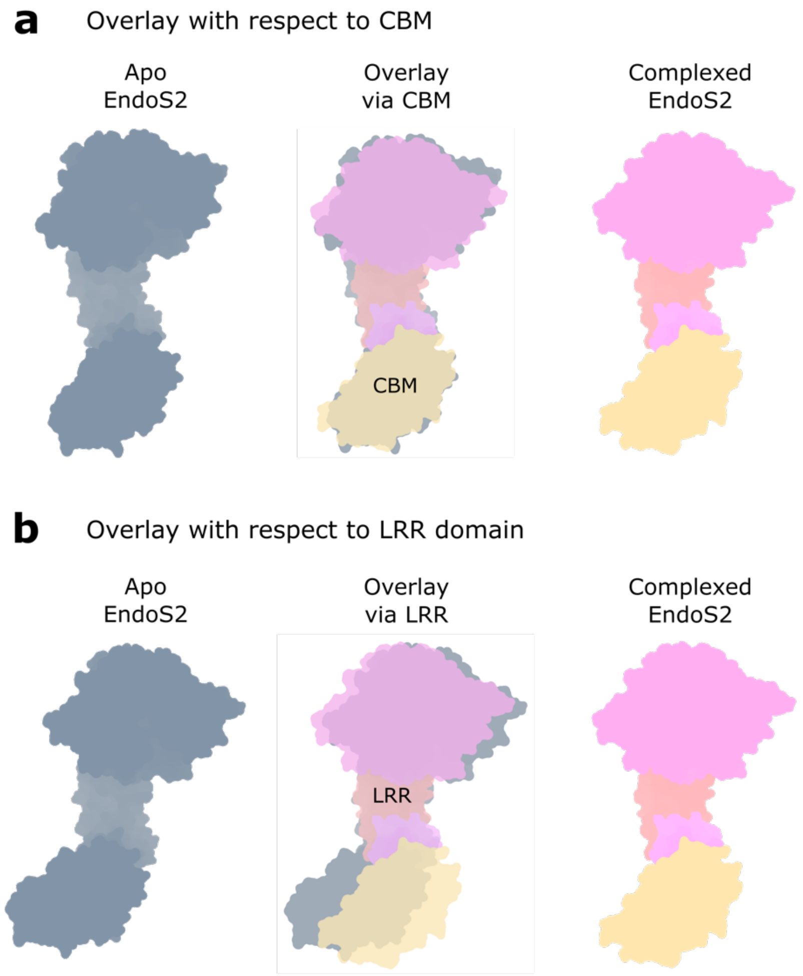

Figure S2: Superposition of EndoS2 from the IgG1 complex with the apo enzyme structure based on **(a)** overlay of the carbohydrate binding module (CBM; calculated by aligning Cα positions for amino acids 681–843) or **(b)** the leucine-rich repeat domain (LRR; calculated by aligning Cα positions for amino acids 387–547). Complexed EndoS2 domains are coloured as in Figure 1; apo EndoS2 (PDB 6E58) is coloured grey.

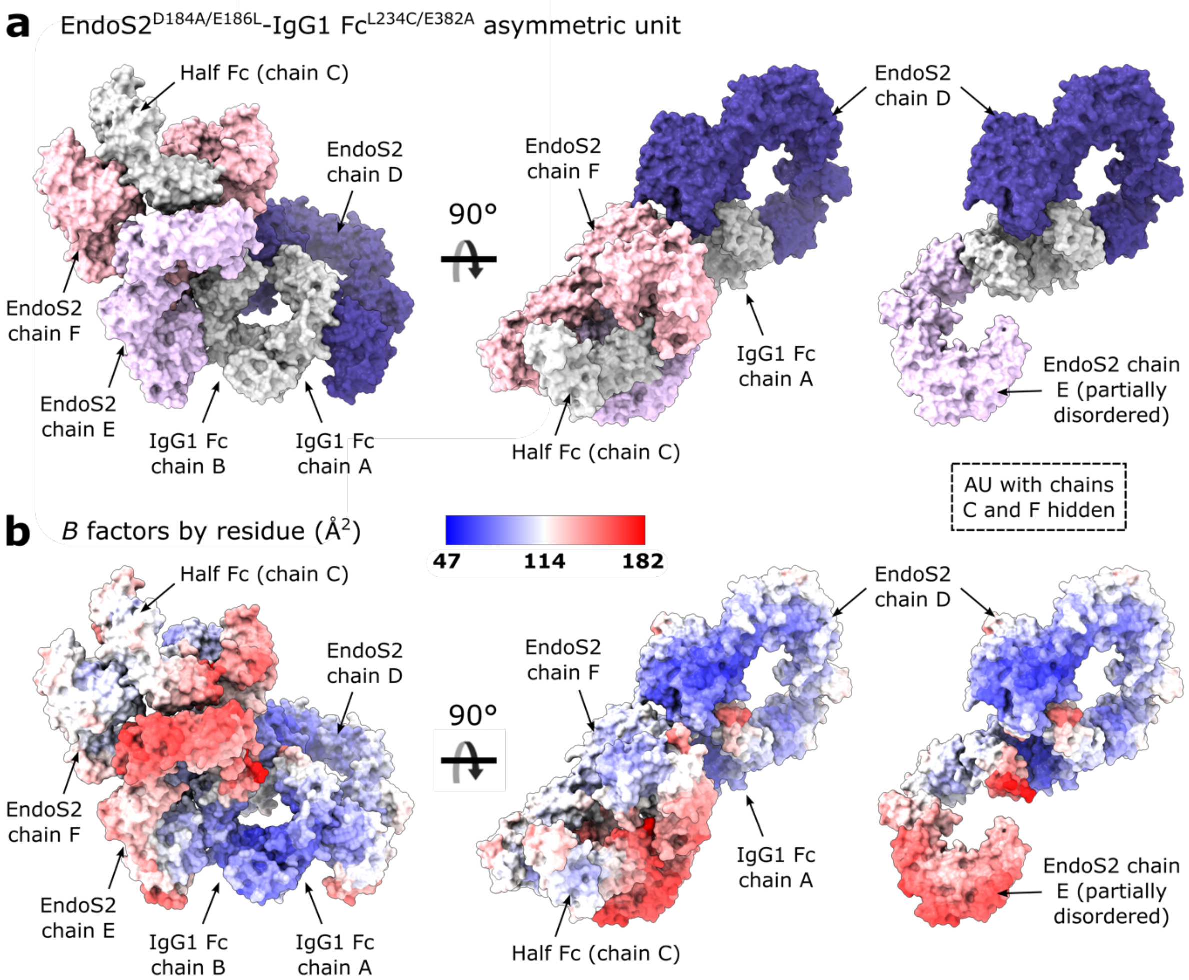

Figure S3: Asymmetric unit (AU) of EndoS2$^{D184A/E186L}$-IgG1 Fc$^{L234C/E382A}$ crystal structure. **a** Arrangement of molecules within the AU, containing three copies of the EndoS2-Fc γ-chain complex. IgG1 Fc$^{L234C/E382A}$ is coloured silver; EndoS2$^{D184A/E186L}$ molecules are coloured purple, lilac and pink. **b** *B* factors per residue. **a, b** Far right panels show the AU with chains C and F hidden for clarity.

Table S2: Average *B* factors per chain for the EndoS2$^{D184A/E186L}$-IgG1 Fc$^{L234C/E382A}$ complex. Average *B* factors are listed for the protein and carbohydrate (if applicable) portions per chain.

Chain within EndoS2-IgG1 Fc complex	Average *B* factors (Å^2) protein / carbohydrate
A (Fc γ-chain)	88.6 / 79.2
B (Fc γ-chain)	93.4
C (Fc γ-chain)	122.8 / 125.2
D (EndoS2)	91.7
E (EndoS2)	131.1
F (EndoS2)	110.0

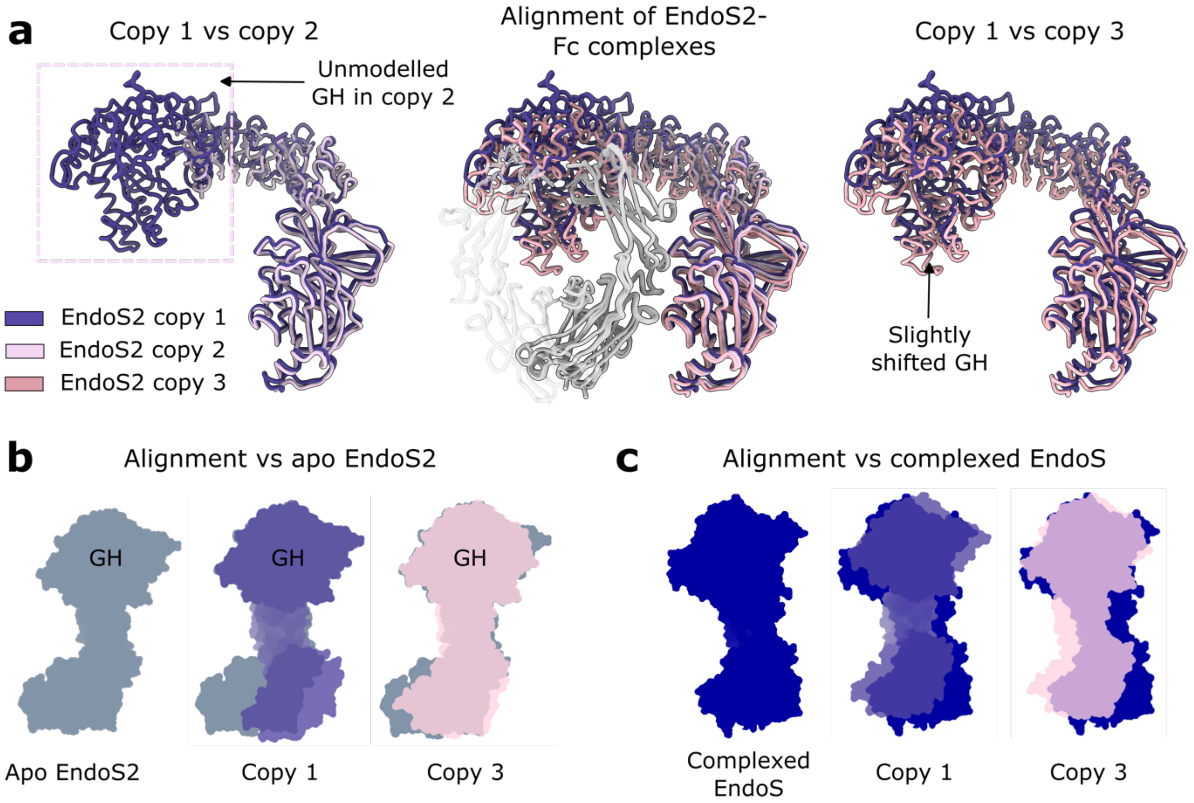

Figure S4: Analysis of the three EndoS2-Fc copies within the asymmetric unit. a Alignment of all three EndoS2-half Fc copies (coloured as in Figure S1a), with copies 1, 2 and 3 comprising EndoS2 chains D, E and F complexed with Fc γ-chains A, B and C, respectively (see Figure S3a). Complexes are aligned by the interfacing Fc Cγ2 domain (amino acids 237–340). EndoS2 copies show some variability in GH domain position; this domain is not modelled in EndoS2 copy 2 due to lack of clear electron density, suggesting flexibility of this domain position. **b** Alignment of EndoS2 copies 1 and 3 against apo EndoS2 (PDB 6E58, coloured grey), via the GH domain (amino acids 46–386) shows rearrangement of the enzyme domains upon complex formation (less pronounced for copy 3). **c** Alignment of EndoS2-Fc copies 1 and 3 against complexed EndoS (PDB 8A49, coloured blue), via the interfacing Fc Cγ2 domain. EndoS2 copies show different domain orientations relative to the Fc, when compared to EndoS. The N-terminal proline rich loop and C-terminal 3-helix bundle (comprising amino acids 98–112 and 924–998, respectively) within EndoS are omitted for clarity. **b**, **c** EndoS2-Fc copy 2 is not analysed due to not being modelled fully.

Table S3: RMSD[1] values for superposition of EndoS2 domains. RMSDs are calculated against other copies of the enzyme in the asymmetric unit (see Table S2 and Figure S1), and versus an apo structure of EndoS2 (PDB 6E58). GH domain analysis is not included for EndoS2 chain E, due to a lack of electron density for this domain.

**Superimposed molecules**		**EndoS2 domain**	**RMSD (Å)**
EndoS2 chain D	EndoS2 chain F	GH[2]	0.236
		LRR[3]	0.698
		hIg[4]	0.343
		CBM[5]	0.178
EndoS2 chain D	EndoS2 chain E	LRR	0.800
		hIg	0.484
		CBM	0.387
EndoS2 chain E	EndoS2 chain F	LRR	0.178
		hIg	0.288
		CBM	0.355
EndoS2 chain D	Unliganded EndoS2	GH	0.345
		LRR	0.887
		hIg	0.498
		CBM	0.597
EndoS2 chain E	Unliganded EndoS2	LRR	0.286
		hIg	0.475
		CBM	0.507
EndoS2 chain F	Unliganded EndoS2	GH	0.379
		LRR	0.350
		hIg	0.472
		CBM	0.527

[1]Root-mean-sqared deviation
[2]Glycosyl hydrolase, amino acids 43–386
[3]Leucine-rich repeat, amino acids 387–547
[4]Hybrid immunoglobulin domain, amino acids 548–680
[5]Carbohydrate-binding module, amino acids 681–843

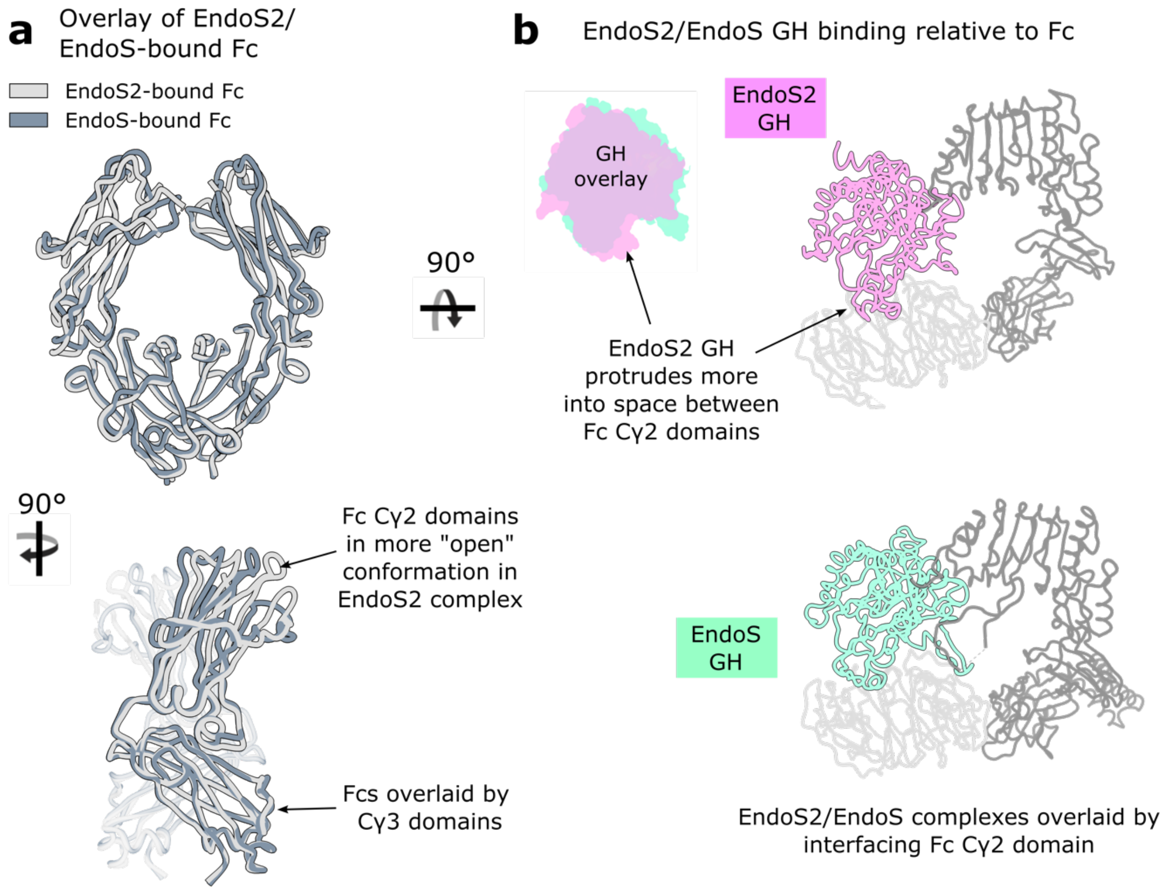

Figure S5: Differential Fc capture by EndoS2/EndoS. a Superposition of EndoS2- and EndoS-bound IgG1 Fc using the Cγ3 domains as reference (calculated by aligning Cα positions for amino acids 341–444). The more "open" conformation of the Fc is apparent from the Cγ2 domains further apart in the EndoS2 complex (silver), compared with the EndoS complex (dark grey). **b** Superposition of EndoS2/EndoS complexes by the interfacing Fc Cγ2 domain (calculated by aligning Cα positions for Fc amino acids 237–340) reveals how the EndoS2 GH domain protrudes further into the space between Fc Cγ2 domains, possibly resulting in the more "open" Fc conformation through displacement of the Cγ2 domains. IgG1 Fcs are depicted in silver, with EndoS2 and EndoS GH domains depicted in pink and cyan, respectively. The remainder of each enzyme is depicted in dark grey. Fc *N*-linked glycans are omitted for clarity.

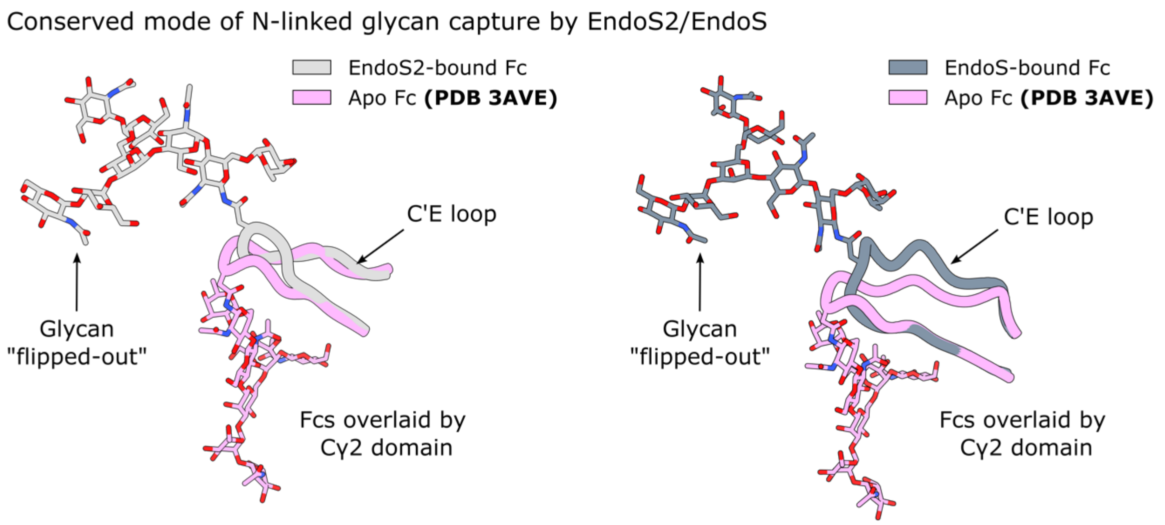

Figure S6: *N*-linked Fc glycan capture by EndoS2/EndoS. Superposition of EndoS2-/EndoS-bound Fc (depicted in silver and dark grey, respectively) with a wild-type IgG1 Fc (PDB 3AVE, depicted in pink) based on the Cγ2 domain (using Cα positions of amino acids 238–340) reveals conformational changes in the C′E loop that support the "flipped-out" conformation of the carbohydrate.

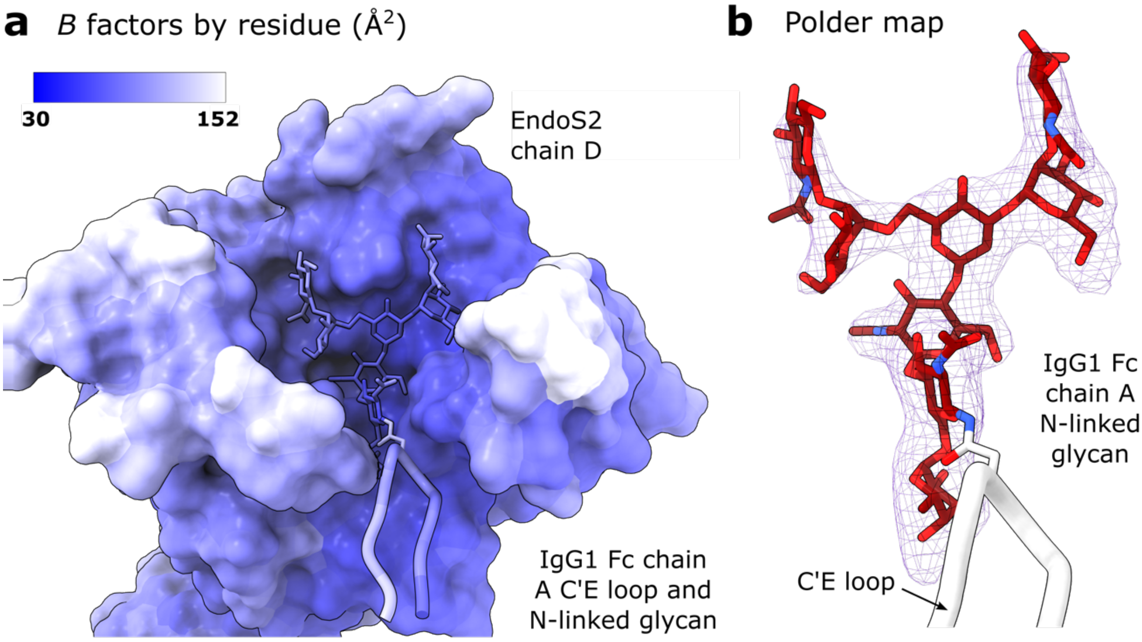

Figure S7: Validation of the *N*-linked glycan conformation in the EndoS2$^{D184A/E186L}$-IgG1 Fc$^{L234C/E382A}$ crystal structure. **a** GH domain shown in surface representation with the Cγ2 domain C′E loop as cartoon and the carbohydrate as sticks, *B* factor coloring from blue to white as indicated. **b** Polder map for the *N*-linked glycan modelled for chain A of IgG1 Fc$^{L234C/E382A}$, calculated using *phenix.polder* (3) in the PHENIX suite using default parameters, shown at 3 σ.

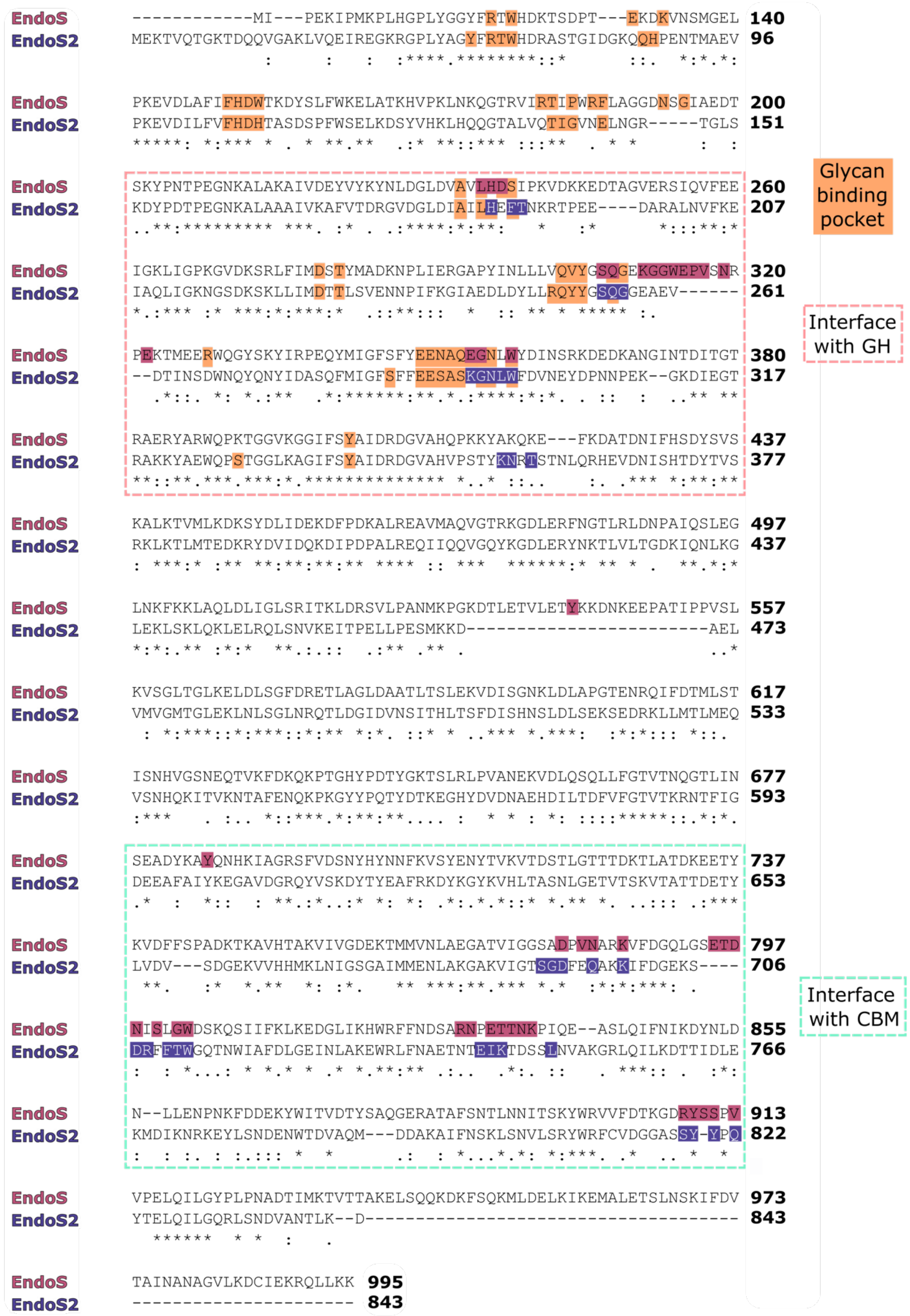

Figure S8: Sequence alignment of EndoS2 and EndoS as generated by ClustalOmega (4). Sequences are shown as present in crystal structures of EndoS2$^{D184A/E186L}$-IgG1 Fc$^{L234C/E382A}$ (PDB 8Q5U) and EndoS-IgG1 Fc (PDB 8A49). Interfaces of EndoS2 and EndoS with the IgG1 Fc protein surface are coloured purple and maroon, respectively, with interfaces formed by the glycosyl hydrolase (GH) domain and carbohydrate-binding module (CBM) of each enzyme indicated with dashed lines. Interfaces of each protein with their glycan substrate are coloured in orange and were calculated using PDBePISA (5).

a

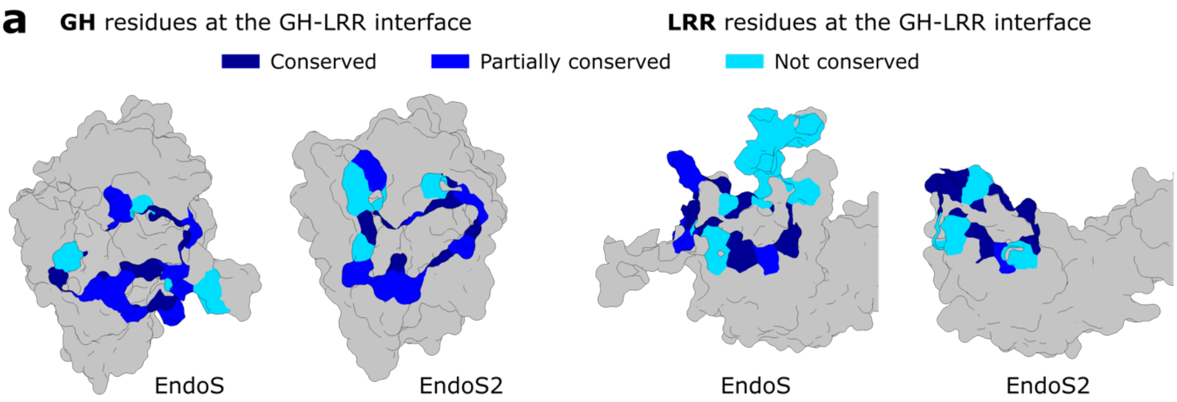

b Multiple sequence alignment

```
EndoS       ------------MI---PEKIPMKPLHGPLYGGYFRTWHDKTSDPT---EKDKVNSMGEL   140
EndoS2      MEKTVQTGKTDQQVGAKLVQEIREGKRGPLYAGYFRTWHDRASTGIDGKQQHPENTMAEV   96
                         :     :   :  :****.********::*      ::.  *:*.*:

EndoS       PKEVDLAFIFHDWTKDYSLFWKELATKHVPKLNKQGTRVIRTIPWRFLAGGDNSGIAEDT   200
EndoS2      PKEVDILFVFHDHTASDSPFWSELKDSYVHKLHQQGTALVQTIGVNELNGR-----TGLS   151
            *****: *:*** * . * **.**  .:* **::*** :::**  . * *       :  :

EndoS       SKYPNTPEGNKALAKAIVDEYVYKYNLDGLDVAVLHDSIPKVDKKEDTAGVERSIQVFEE   260
EndoS2      KDYPDTPEGNKALAAAIVKAFVTDRGVDGLDIAILHEFTNKRTPEE----DARALNVFKE   207
            ..**:********* ***. :* . .:****:*:**:   *   :*       *:::**:*

EndoS       IGKLIGPKGVDKSRLFIMDSTYMADKNPLIERGAPYINLLLVQVYGSQGEKGGWEPVSNR   320
EndoS2      IAQLIGKNGSDKSKLLIMDTTLSVENNPIFKGIAEDLDYLLRQYYGSQGGEAEV------   261
            *.:*** :* ***:*:***:*  .::**::: *  :: ** * ***** :.

EndoS       PEKTMEERWQGYSKYIRPEQYMIGFSFYEENAQEGNLWYDINSRKDEDKANGINTDITGT   380
EndoS2      --DTINSDWNQYQNYIDASQFMIGFSFFEESASKGNLWFDVNEYDPNNPEK--GKDIEGT   317
              .*::. *: *.:**  .*:******:**.*.:****:*:*. . ::  :  ..** **

EndoS       RAERYARWQPKTGGVKGGIFSYAIDRDGVAHQPKKYAKQKE---FKDATDNIFHSDYSVS   437
EndoS2      RAKKYAEWQPSTGGLKAGIFSYAIDRDGVAHVPSTYKNRTSTNLQRHEVDNISHTDYTVS   377
            **::**.***.***:*.************** *..* ::..    :. .*** *:**:**

EndoS       KALKTVMLKDKSYDLIDEKDFPDKALREAVMAQVGTRKGDLERFNGTLRLDNPAIQSLEG   497
EndoS2      RKLKTLMTEDKRYDVIDQKDIPDPALREQIIQQVGQYKGDLERYNKTLVLTGDKIQNLKG   437
            : ***:* :** **:**:**:** **** :: ***  ******:* ** *  .  **.*:*

EndoS       LNKFKKLAQLDLIGLSRITKLDRSVLPANMKPGKDTLETVLETYKKDNKEEPATIPPVSL   557
EndoS2      LEKLSKLQKLELRQLSNVKEITPELLPESMKKD------------------------AEL   473
            *:*:.** :*:*  **.:.::  .:** .**  .                        ..*
```

Figure S9: Conservation of EndoS/EndoS2 GH domain interface with LRR–hIg–CBM scaffold. a Surface representation of GH and LRR domains from complexed EndoS/EndoS2, with residues present at their respective interdomain GH–LRR interface coloured (dark blue, conserved; medium blue, partially conserved; light blue, not conserved). **b** Multiple sequence alignment of EndoS and EndoS2 generated by ClustalOmega (4). Residues present at the GH–LRR interface for each enzyme are coloured as in panel **a**. Intefacing residues were calculated using PDBePISA (5).

a IgG1 Fc$^{L234C/E382A}$ (residues 221-447)

DKTHTCPPCPAPE**C**LGGPSVFLFPPKPKDTLMISRTPEVTCVVVDVSHEDPEVKFNW
YVDGVEVHNAKTKPREEQYNSTYRVVSVLTVLHQDWLNGKEYKCKVSNKALPAPIE
KTISKAKGQPREPQVYTLPPSREEMTKNQVSLTCLVKGFYPSDIAVEW**A**SNGQPEN
NYKTTPPVLDSDGSFFLYSKLTVDKSRWQQGNVFSCSVMHEALHNHYTQKSLSLSP
GK

b EndoS2$^{D184A/E186L}$ (residues 38-843)

MEKTVQTGKTDQQVGAKLVQEIREGKRGPLYAGYFRTWHDRASTGIDGKQQHPEN
TMAEVPKEVDILFVFHDHTASDSPFWSELKDSYVHKLHQQGTALVQTIGVNELNGR
TGLSKDYPDTPEGNKALAAAIVKAFVTDRGVDGLDI**AIL**HEFTNKRTPEEDARALNV
FKEIAQLIGKNGSDKSKLLIMDTTLSVENNPIFKGIAEDLDYLLRQYYGSQGGEAEV
DTINSDWNQYQNYIDASQFMIGFSFFEESASKGNLWFDVNEYDPNNPEKGKDIEG
TRAKKYAEWQPSTGGLKAGIFSYAIDRDGVAHVPSTYKNRTSTNLQRHEVDNISHT
DYTVSRKLKTLMTEDKRYDVIDQKDIPDPALREQIIQQVGQYKGDLERYNKTLVLTG
DKIQNLKGLEKLSKLQKLELRQLSNVKEITPELLPESMKKDAELVMVGMTGLEKLNL
SGLNRQTLDGIDVNSITHLTSFDISHNSLDLSEKSEDRKLLMTLMEQVSNHQKITVK
NTAFENQKPKGYYPQTYDTKEGHYDVDNAEHDILTDFVFGTVTKRNTFIGDEEAFAI
YKEGAVDGRQYVSKDYTYEAFRKDYKGYKVHLTASNLGETVTSKVTATTDETYLVDV
SDGEKVVHHMKLNIGSGAIMMENLAKGAKVIGTSGDFEQAKKIFDGEKSDRFFTW
GQTNWIAFDLGEINLAKEWRLFNAETNTEIKTDSSLNVAKGRLQILKDTTIDLEKMD
IKNRKEYLSNDENWTDVAQMDDAKAIFNSKLSNVLSRYWRFCVDGGASSYYPQYT
ELQILGQRLSNDVANTLKDLLEHHHHHH

Figure S10: Constructs used for EndoS2$^{D184A/E186L}$-IgG1 $^{FcL234C/E382A}$ expression and purification. **a** IgG1 Fc containing L234C and E382A exchanges. **b** Truncated EndoS2 (residues 38–843) containing D184A and E186L exchanges to abolish catalytic activity. Amino acid variations are highlighted in orange; C-terminal linker and His-tag in EndoS2 construct is highlighted in blue.

Table S4: TLS parameters used in refinement of EndoS2$^{D184A/E186L}$-IgG1 Fc$^{L234C/E382A}$ crystal structure.

EndoS2$^{D184A/E186L}$	IgG1 Fc$^{L234C/E382A}$
TLS For Peptide chain D<br>RANGE ′D 45. ′ ′D 386. ′<br>RANGE ′D 387. ′ ′D 547. ′<br>RANGE ′D 548. ′ ′D 680. ′<br>RANGE ′D 681. ′ ′D 832. ′	TLS For Peptide chain A<br>RANGE ′A 237. ′ ′A 340. ′<br>RANGE ′A 341. ′ ′A 444. ′
TLS For Peptide chain E<br>RANGE ′E 264. ′ ′E 386. ′<br>RANGE ′E 387. ′ ′E 547. ′<br>RANGE ′E 548. ′ ′E 680. ′<br>RANGE ′E 681. ′ ′E 832. ′	TLS For Peptide chain B<br>RANGE ′B 237. ′ ′B 340. ′<br>RANGE ′B 341. ′ ′B 444. ′
TLS For Peptide chain F<br>RANGE ′F 46. ′ ′F 386. ′<br>RANGE ′F 387. ′ ′F 547. ′<br>RANGE ′F 548. ′ ′F 680. ′<br>RANGE ′F 681. ′ ′F 832. ′	TLS For Peptide chain C<br>RANGE ′C 236. ′ ′A 340. ′<br>RANGE ′C 341. ′ ′C 443. ′

Supplementary methods

Cloning. The gene fragment encoding for IgG1 Fc was cloned into a pFUSE vector, introducing a mutation for an E382A exchange, as described previously (2). A further mutation was introduced to encode a L234C exchange, in order to introduce an additional hinge disulphide and thus reduce Fc hinge flexibility for improved crystallisation (Figure S10).

The enzyme EndoS2$_{38–843}$ was expressed with an C-terminal linker and His tag (sequence LEHHHHHH, as described previously (6)) (Figure S10). The amino acid exchange variant D184A/E186L was used in order to inactivate the enzyme (genome accession number ACI61688.1, cloned into a pET21a(+) vector by NBS Biologicals).

Protein expression. IgG1 Fc$^{L234C/E382A}$ was transiently expressed in Freestyle293F cells (ThermoFisher), using FreeStyle™ MAX Reagent (ThermoFisher). Cells were incubated at 37 °C, 8% CO_2, shaking at 125 rpm (New Brunswick S41i incubator). Cell supernatants were harvested after seven days by centrifugation at 3100 × *g* for 30 minutes and filtered through a Nalgene™ Rapid-Flow™ Sterile Disposable Filter Unit (0.2 µm; ThermoFisher).

EndoS2$^{D184A/E186L}$ was expressed in BL21 (DE3)pLysS cells (ThermoFisher). Cells were grown in the presence of 100 µg/mL ampicillin and 34 µg/mL chloramphenicol in Terrific Broth (Melford), until reaching an OD_{600} of 0.8, at which 1 mM IPTG was added. Cells were left to shake at 200 rpm (Innova 43 R incubator, New Brunswick Scientific) overnight at 25 °C. Cells were harvested by 20 minutes of centrifugation at 6220 × *g*.

Protein purification. The IgG1 Fc protein was purified by affinity purification with a HiTrap Protein A HP column (Cytiva). Eluted antibody was concentrated in a Vivaspin 20 Centrifgual Concentrator (MWCO 30 kDa; Sigma).

For EndoS2, harvested bacterial cell pellets were resuspended in PBS containing 2 µg/mL DNAse1 (Sigma) and a pinch of lysozyme (Sigma). Cells were broken with a cell disruptor (Constant Cell Disruption Systems), removing cell debris / membranes by harvesting centrifugation at 3100 × *g* for 20 minutes, followed by clearing centrifugation at 100,000 × *g* for 60 minutes. Filtered supernatant (Nalgene™ Rapid-Flow™ Sterile Disposable Filter Unit, 0.2 µm; ThermoFisher) was applied to a 5 mL HisTrap HP column (Cytiva), eluted, concentrated, and further purified using size exclusion chromatography on a Superdex 200 16/600 (Cytiva) equilibrated with 10 mM HEPES, 150 mM NaCl, pH 8.0. Fractions

corresponding to the main peak were pooled and concentrated in a Vivaspin 20 Centrifugal Concentrator (MWCO 50 kDa; Sigma).

EndoS2-IgG1 Fc complex formation. Protein concentrations of EndoS2$^{D184A/E186L}$ and IgG1 Fc$^{L234C/E382A}$ were determined using a DS-11+ Spectrophotometer (DeNovix), using molecular weight and extinction coefficient values provided by the ProtParam tool (7). EndoS2$^{D184A/E186L}$ and IgG1 Fc$^{L234C/E382A}$ were combined in a 2:1 molar ratio and the resulting complex applied to a Superdex 200 16/100 column (Cytiva) equilibrated in 10 mM HEPES, 150 mM NaCl, pH 8.0. Fractions corresponding to the main peak were pooled.

Crystallisation. The purified complex was subsequently exchanged into a buffer containing 50 mM HEPES, 150 mM KCl, pH 7.5, and concentrated to 10 mg/mL prior to crystallisation, using a Vivaspin 20 Centrifugal Concentrator (MWCO 30 kDa; Sigma). Crystal trays were set up using an Oryx4 robot (Douglas Instruments) and grown at 21 °C using sitting drop vapour diffusion in 0.1 M carboxylic acids, 0.1 M buffer system 3, pH 8.5, precipitant mix 4 (condition G12 in Morpheus crystallisation screen (8), Molecular Dimensions).

Structure determination. Crystals were flash-frozen in liquid nitrogen. Data collection was carried out on beamline I03 at Diamond Light Source (Oxfordshire, UK) at 100 K with a wavelength of 0.9763 Å. Diffraction images were processed using DIALS (9). The structure was solved by molecular replacement with the program Molrep (10) within the ccp4i2 suite (11), using 3AVE and 6E58 search models for IgG1 Fc and EndoS2, respectively. The model was improved using successive rounds of manual model building and refinement, using Coot (12) and Refmac5 (13), respectively. Refinement was carried out using local non-crystallographic symmetry restraints, using translation-libration-screw groups (Table S4) and restraints generated from PDB-REDO (14). Electron density maps were calculated using map sharpening in Refmac5 (13). The Coot carbohydrate module (15) and Privateer (16) were used to build and validate *N*-linked glycan structure.

Supplementary references:

1. Williams, C. J., Headd, J. J., Moriarty, N. W., Prisant, M. G., Videau, L. L., Deis, L. N., Verma, V., Keedy, D. A., Hintze, B. J., Chen, V. B., Jain, S., Lewis, S. M., Arendall, W. B., Snoeyink, J., Adams, P. D., Lovell, S. C., Richardson, J. S., and Richardson, D. C. (2018) MolProbity: more and better reference data for improved all-atom structure validation. *Protein Sci* **27**, 293-315
2. Sudol, A. S. L., Butler, J., Ivory, D. P., Tews, I., and Crispin, M. (2022) Extensive substrate recognition by the streptococcal antibody-degrading enzymes IdeS and EndoS. *Nat Commun* **13**, 7801
3. Liebschner, D., Afonine, P. V., Moriarty, N. W., Poon, B. K., Sobolev, O. V., Terwilliger, T. C., and Adams, P. D. (2017) Polder maps: improving OMIT maps by excluding bulk solvent. *Acta Crystallogr D Struct Biol* **73**, 148-157
4. Sievers, F., Wilm, A., Dineen, D., Gibson, T. J., Karplus, K., Li, W., Lopez, R., McWilliam, H., Remmert, M., Söding, J., Thompson, J. D., and Higgins, D. G. (2011) Fast, scalable generation of high-quality protein multiple sequence alignments using Clustal Omega. *Mol Syst Biol* **7**, 539
5. Krissinel, E., and Henrick, K. (2007) Inference of macromolecular assemblies from crystalline state. *J Mol Biol* **372**, 774-797
6. Klontz, E. H., Trastoy, B., Deredge, D., Fields, J. K., Li, C., Orwenyo, J., Marina, A., Beadenkopf, R., Günther, S., Flores, J., Wintrode, P. L., Wang, L. X., Guerin, M. E., and Sundberg, E. J. (2019) Molecular basis of broad spectrum *N*-glycan specificity and processing of therapeutic IgG monoclonal antibodies by endoglycosidase S2. *ACS Cent Sci* **5**, 524-538
7. Wilkins, M. R., Gasteiger, E., Bairoch, A., Sanchez, J. C., Williams, K. L., Appel, R. D., and Hochstrasser, D. F. (1999) Protein identification and analysis tools in the ExPASy server. *Methods Mol Biol* **112**, 531-552
8. Gorrec, F. (2009) The MORPHEUS protein crystallization screen. *J Appl Crystallogr* **42**, 1035-1042
9. Winter, G., Waterman, D. G., Parkhurst, J. M., Brewster, A. S., Gildea, R. J., Gerstel, M., Fuentes-Montero, L., Vollmar, M., Michels-Clark, T., Young, I. D., Sauter, N. K., and Evans, G. (2018) DIALS: implementation and evaluation of a new integration package. *Acta Crystallogr D Struct Biol* **74**, 85-97
10. Vagin, A., and Teplyakov, A. (2010) Molecular replacement with MOLREP. *Acta Crystallogr D Biol Crystallogr* **66**, 22-25
11. Potterton, L., Agirre, J., Ballard, C., Cowtan, K., Dodson, E., Evans, P. R., Jenkins, H. T., Keegan, R., Krissinel, E., Stevenson, K., Lebedev, A., McNicholas, S. J., Nicholls, R. A., Noble, M., Pannu, N. S., Roth, C., Sheldrick, G., Skubak, P., Turkenburg, J., Uski, V., von Delft, F., Waterman, D., Wilson, K., Winn, M., and Wojdyr, M. (2018) CCP4i2: the new graphical user interface to the CCP4 program suite. *Acta Crystallogr D Struct Biol* **74**, 68-84
12. Emsley, P., Lohkamp, B., Scott, W. G., and Cowtan, K. (2010) Features and development of Coot. *Acta Crystallogr D Biol Crystallogr* **66**, 486-501
13. Murshudov, G. N., Skubák, P., Lebedev, A. A., Pannu, N. S., Steiner, R. A., Nicholls, R. A., Winn, M. D., Long, F., and Vagin, A. A. (2011) REFMAC5 for the refinement of macromolecular crystal structures. *Acta Crystallogr D Biol Crystallogr* **67**, 355-367
14. Joosten, R. P., Long, F., Murshudov, G. N., and Perrakis, A. (2014) The PDB_REDO server for macromolecular structure model optimization. *IUCrJ* **1**, 213-220
15. Emsley, P., and Crispin, M. (2018) Structural analysis of glycoproteins: building *N*-linked glycans with Coot. *Acta Crystallogr D Struct Biol* **74**, 256-263

16. Agirre, J., Iglesias-Fernández, J., Rovira, C., Davies, G. J., Wilson, K. S., and Cowtan, K. D. (2015) Privateer: software for the conformational validation of carbohydrate structures. *Nat Struct Mol Biol* **22**, 833-834
